# Telehomecare as a Catalyst for a Multifaceted Transformation Towards Sustainable Practices: A Qualitative Study From a Practical Nurses' Perspective

**DOI:** 10.1111/jan.17072

**Published:** 2025-05-23

**Authors:** Rydgren Melanie, Andtfolk Malin, Anåker Anna, Estman Linda, Fagerström Lisbeth

**Affiliations:** ^1^ Department of Health Sciences Faculty of Education and Welfare Studies, Åbo Akademi University Vaasa Finland; ^2^ School of Health and Welfare Dalarna University Falun Sweden; ^3^ Department of Caring and Ethics The Faculty of Health Sciences, University of Stavanger Stavanger Norway

**Keywords:** climate change, digital health technology, health care delivery, homecare services, nurses, nursing, practical nurses, sustainability, telehomecare, telenursing

## Abstract

**Aim(s):**

To explore how practical nurses perceive telehomecare in relation to sustainability.

**Design:**

This study had a qualitative exploratory design.

**Methods:**

Ten practical nurses working with telehomecare were interviewed in February–April 2024. The interviews were individual, semi‐structured and were analysed through Braun and Clarke's reflexive thematic analysis.

**Results:**

The overarching theme of this study was ‘Telehomecare: A catalyst for a multifaceted transformation towards sustainable practices in homecare nursing’, with three main themes and seven subthemes. The three main themes were ‘Reshaped delivery of care’, ‘Reformed work environment’ and ‘Reallocated resources'. The findings reveal that nurses have multifaceted perspectives on telehomecare in relation to sustainability, recognising both its positive and negative impacts on healthcare organisations, nurses and clients while also acknowledging that more sustainable practices demand significant changes in the healthcare environment.

**Conclusion:**

Telehomecare has significant impacts on multiple dimensions of sustainability within healthcare and notable drawbacks. These findings emphasise the critical need for comprehensive education and training in sustainable digital work practices to enhance healthcare professionals' awareness of environmental impacts. This underscores the importance of transformative leadership that drives organisational change towards sustainable healthcare practices and implements effective sustainability policies.

**Impact:**

The findings present some aspects of telehomecare that contribute to a lesser environmental impact from a nursing care perspective, encouraging healthcare leaders to make conscious and effective strategic decisions towards more sustainable healthcare. The findings strengthen nurses, leaders and policymakers' knowledge and awareness of sustainable nursing activities in the digital milieu, highlighting the urgent need for transformation of healthcare practices to decrease the environmental impact.

**Reporting Method:**

The study followed the consolidated criteria for reporting qualitative studies (COREQ).

**Patient or Public Contribution:**

This study did not include patient or public involvement in its design, conduct or reporting.

**Summary**

- Why is this research or review needed?
  ○ Healthcare as a sector is committed to the well‐being of populations but paradoxically contributes significantly to environmental degradation, making the sustainability of healthcare systems a critical concern.
  ○ Limited research has focused on practical nurses' experiences of and insights into the sustainability of digital healthcare services, particularly in the context of homecare.
  ○ Research on the sustainability of technologies in healthcare is limited, particularly with respect to their unintended environmental or social consequences.
- What are the key findings?
  ○ Telehomecare can have a positive effect on the environmental, economic and social sustainability of healthcare organisations, although its limitations must be accounted for.
  ○ Telehomecare has the potential to transform nursing activities and work environments, and the well‐being of healthcare personnel as key for a viable, sustainable workforce.
  ○ There is a need for education and training in sustainable digital practices for healthcare professionals, leaders and policymakers as well as more sustainable coordination and collaboration within healthcare organisations.
- How should the findings be used to influence policy/practice/research/education?
  ○ The findings could be used to guide the adoption of telehomecare services through strategies that address social, economic and environmental concerns.
  ○ The findings could be used in training and education to equip healthcare professionals with the knowledge needed to perform sustainable digital healthcare.
  ○ The findings could support healthcare leaders in adopting sustainable practices, fostering collaboration and improving workplace conditions.
- What does this paper contribute to the wider global clinical community?
  ○ Telehomecare can have a positive effect on environmental, economical and social sustainability of healthcare organisations, although its limitations must be accounted for.
  ○ Telehomecare has the potential to transform nursing activities and work environments, as well as the well‐being of healthcare personnel, as key for a viable, sustainable workforce.
  ○ There is a need for comprehensive education and training in sustainable digital work practices for healthcare professionals, leaders and policymakers to strengthen knowledge and awareness as well as more sustainable coordination and collaboration within healthcare organisations.

## 1 Introduction

Initiatives for a more sustainable healthcare have become more frequent in recent years in response to the increasing effects of climate change. As more pressure is added to the healthcare sector due to an increasing need for healthcare services, an aging population and lack of healthcare personnel, the sustainability of healthcare systems has become a critical concern (World Health Organization [WHO] 2020). The healthcare sector is committed to the well‐being of populations but paradoxically contributes to environmental degradation, being responsible for 4%–6% of global net greenhouse gas emissions in 2020 (van Daalen et al. 2024). In Finland, the social and healthcare sector accounted for 6.5% of the country's net emissions in 2019 (Pulkki et al. 2023, p. 30), emphasising the need for carbon‐neutral practices. The concept of sustainability has environmental, economic and social dimensions (Purvis et al. 2019), and the practices and principles of sustainable healthcare—including ill health prevention, client self‐management, streamlined service delivery, low‐carbon alternatives and efficient resource use (Mortimer 2010)—are becoming critical frameworks for mitigating healthcare's environmental footprint and enabling a viable healthcare system. Yet, implementing these initiatives faces challenges related to cost, regulations and organisational inertia (Aboueid et al. 2023).

The need for more sustainable practices requires significant changes in the healthcare milieu (WHO 2020). Nurses, such as practical nurses, play a central role in advancing sustainable healthcare by promoting preventive care and advocating for resource‐efficient practices. Practical nurses in Finland complete a vocational education focused on primary care practices. Practical nurses in homecare assist clients with hygiene, nutrition and medication and can perform measurements and assessments of the client's well‐being. Their frontline presence in healthcare delivery enables them to implement sustainable interventions directly in patient care (Cruz et al. 2024). Telehomecare, a form of homecare performed remotely through technologies such as video communication (Hoffrén‐Mikkola et al. 2024), represents a pathway towards more sustainable practices. Delivering care remotely can optimise resource use (Muschol et al. 2022) while posing challenges related to financial investment, energy demands and the generation of e‐waste (Ravindrane and Patel 2022). Furthermore, research on the sustainability of technologies in healthcare is limited, particularly with respect to their unintended environmental or social consequences (Serra et al. 2022).

### 1.1 Background

The WHO (2021) advocates for the integration of digital technologies, such as information and communications technologies, to foster sustainable practices within healthcare organisations. In the context of homecare, telenursing and ‐medicine involve the use of video‐communication devices, remote monitoring systems and other technologies to provide care at a distance. This form of service delivery can be referred to as telehomecare (Hoffrén‐Mikkola et al. 2024). Telenursing and ‐medicine enhance the accessibility of healthcare services (Björndell and Premberg 2021), improve resource efficiency (Muschol et al. 2022) and contribute to better patient satisfaction and patient outcomes (Liang et al. 2021; Orlando et al. 2019). Additionally, healthcare professionals performing telehomecare have been reported to experience an increase in well‐being and positive effects on their work situation (Björndell and Premberg 2021). From an environmental perspective, telenursing and ‐medicine reduce travel‐related healthcare emissions (Serra et al. 2022) and the use of personal protective equipment (Candel et al. 2021), although there is evidence of increased electricity usage associated with digital healthcare technologies (Holmner et al. 2014). Purohit et al. (2021) note that the environmental impact of telemedicine equipment is minimal compared to that of travel emissions, but few studies have focused on nurses' travel to clients' homes (Donald and Irukulla 2022). However, delivering nursing care remotely presents several challenges, as effective telenursing requires a robust technical infrastructure to ensure data integrity and security (Smith et al. 2020).

Many previous studies concerning the environmental sustainability of telenursing apply a quantitative approach and lack nuance and an in‐depth understanding of sustainable digital nursing practices (Purohit et al. 2021), particularly in the context of homecare (Koltsida and Jonasson 2021). This gap underscores the need for further research to explore practical nurses' subjective understanding of the sustainability of telehomecare through a qualitative approach. Such research can provide deeper insights into how technologies can be effectively integrated into everyday nursing activities and whether they reduce the environmental impact of nursing care.

## 2 The Study

### 2.1 Aim

The aim of this study was to explore how practical nurses perceive telehomecare in relation to sustainability. The research question was the following: how do practical nurses experience the contribution of telehomecare to the transformation towards sustainable practices?

### 2.2 Design

This study applied a qualitative exploratory design to capture and interpret the lived experiences of the study participants (Polit and Beck 2011). The study used semi‐structured, individual interviews, analysed through reflexive thematic analysis (Braun and Clarke 2021), as the flexibility of this method yields an in‐depth understanding of the participants' perspectives. The study employed an inductive analysis technique and followed the consolidated criteria for reporting qualitative studies (COREQ) (Tong et al. 2007).

### 2.3 Study Setting

In Finland, there are 21 regional, self‐governing well‐being services counties responsible for organising healthcare, social welfare and rescue services. The study was conducted in three bilingual municipalities in a well‐being services county in Finland that covers both urban and rural areas. Homecare is provided to individuals whose functional capacity has diminished due to aging, illness, injury or related conditions (Social Welfare Act, 2014). In the well‐being services county, the practical nurses use their own cars to travel between clients and the office and are reimbursed the cost of the fuel used. To improve the effectiveness and quality of care, telehomecare services have recently been introduced as part of homecare services. Telehomecare services in Finland are delivered by practical nurses who have completed a comprehensive three‐year vocational education programme.

Nursing tasks that do not require the physical presence of the nurse—including follow‐up on clients' well‐being, supervision of blood sugar and blood pressure measurements, nutritional assessments and medication—are performed remotely through telehomecare. The practical nurse calls the client on a two‐way video‐communication device (tablet) installed in the client's home. The digital visits are approximately 10 min long, depending on the needs of the clients and are pre‐determined and agreed upon with the client when the service starts.

Healthcare personnel from the hospital discharge team assess a client's status and willingness to participate in the telehomecare service before the service is started. Clients receiving telehomecare are required to be somewhat independent and not have grave cognitive illnesses, as the client's ability to undertake activities is a vital part of the remote service. No digital competence is required from the client, as the nurse activates the communication device remotely.

### 2.4 Recruitment and Participants

Recruitment was performed with a purposive sample. The researchers were provided with the e‐mail addresses of the participants by healthcare leaders. Initial contact with participants was made via email by the first author, inviting practical nurses to participate in the study. Ten practical nurses agreed to participate. Participants were provided with detailed information on the study's objectives, research methods, data management plan and data protection notice. One practical nurse declined participation. After ten interviews, information power was achieved, as the participants were well‐aligned with the research focus, and the data were rich and qualitatively robust (Malterud et al. 2016).

The inclusion criteria were practical nurses working in telehomecare for a minimum of three months, and a minimum of three months work experience in traditional, physical homecare visits and/or care services for older adults. The inclusion criteria were selected to ensure participants' knowledge and experience in the chosen context. All the participants were actively working in telehomecare services during the data collection period. Characteristics of participants are presented in Table 1.

**TABLE 1:** Characteristics of participants.

Characteristics (*n* = 10)	Median	Range	Division
Demographics			
Age (years)	44.5	28.0–64.0	
Gender F/M^a^			10/0
Language F/S^b^			4/6
Years of experience			
In nursing	20.0	0.8–42.0	
In telehomecare	0.7	0.4–7.0	

^a^Female/Male.

^b^Finnish/Swedish.

### 2.5 Data Collection

Healthcare leaders, practical nurses working in telehomecare, and the hospital discharge team were consulted prior to the study to gain more information on the telehomecare services and the working conditions. One operational unit was visited by the first author prior to data collection to gain insight on the working conditions. The semi‐structured interview guide, consisting of nine questions, was developed according to Kallio et al. (2016). The guide was based on the purpose of the study and developed by reviewing previous research and relevant literature. Three of the nine questions were included in this study, as not all the collected material from the interviews was utilised. The authors collaborated in the development of the interview guide questions. The interview guide was not piloted, as the flexible and semi‐structured nature of the interviews allowed for real‐time adjustments to the guide based on participant responses, mitigating the need for a formal pilot. The themes in the interview guide concerned sustainable healthcare and the telehomecare service. The interview questions were the following: ‘Why has the telehomecare service been introduced in your organization and what are your experiences with the service? Comparing remote care visits through the telehomecare service to physical care visits, what are the advantages and disadvantages? How does the telehomecare service support and/or hinder you from working sustainably?’ The interview guide was approved by the well‐being services county where the study was performed.

The data collection was conducted during February–April in 2024. All the interviews were performed by the first author. Participants got the option to choose between face‐to‐face and digital interviews. All the participants chose to be interviewed digitally. Participants' informed consent was obtained orally prior to each interview.

The interviews were performed through video communication, in both official languages in Finland (Finnish, Swedish). Before each interview, the dimensions of sustainability (Purvis et al. 2019) were introduced to assure mutual understanding. Each interview was video‐ and audio‐recorded and lasted 30–60 min. Non‐verbal behaviours were noted to reinforce the meaning units and ensure accurate understanding of the participants' responses. No participant withdrew their participation during the research process.

### 2.6 Ethical Considerations

This study followed the principles of good research practices as outlined by the Finnish National Board on Research Integrity TENK (2023). The study was approved by the well‐being services county prior to data collection (1538/13.01/2023). Participants were provided with detailed information about the study, including its purpose, research methods and data management, through e‐mail and orally prior to the interviews. Participants were informed that participation was voluntary, and they had the option to withdraw their participation at any time during the research process. Confidentiality was assured, and participants were coded with numbers to protect their privacy. Informed consent was obtained from all participants before the interviews began.

### 2.7 Data Analysis

The researcher who collected the data transcribed the recordings of the interviews and noted the non‐verbal behaviours of the participants. The recordings were listened to multiple times to ensure correct transcription, which reduced the risk of oversight. The transcribed interviews were numbered, and no identifiable features were transcribed to ensure confidentiality. Member checking was not carried out in this study.

**FIGURE 1:**
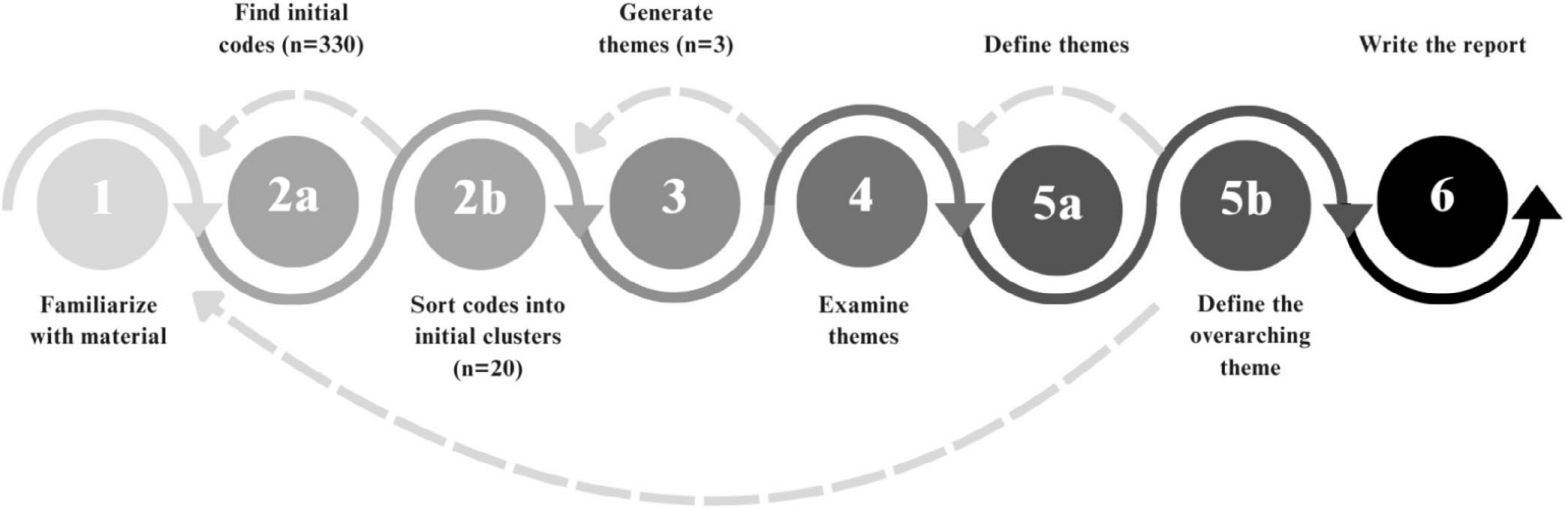
Illustrated process of the thematic reflexive analysis by Braun and Clarke (2021).

The data were analysed in six steps through reflexive thematic analysis (Braun and Clarke 2021). Figure 1 was developed to illustrate the process of the analysis.

The researchers familiarised themselves with the material individually by reading the transcriptions multiple times while taking notes. Key meaningful units that correspond to the research question were highlighted and extracted into a joint document by the first author. The meaningful units were carefully analysed iteratively by four researchers. Both semantic and latent codes were created manually. The researchers then formed themes by cooperatively sorting the codes into clusters to strengthen the validity of the study. The clusters were hierarchically organised in a table and then adjusted according to similarities in the codes and clusters. The final table comprised 12 clusters, which were combined and developed into three themes. Clusters irrelevant to the aim of the study were extracted and stored in another table. The researchers adjusted and formed the three themes and developed seven subthemes that visualise the collected data. An overarching theme was developed based on the main themes and subthemes. Lastly, all the authors examined and defined the formed themes in collaboration, and quotes were extracted by the first author to visualise the findings.

### 2.8 Rigour

The rigour in this study was considered according to the trustworthiness criteria in qualitative research by Lincoln and Guba (1985). The first author who performed the data collection and transcription of the interviews was a registered nurse with experience in physical homecare, which had a positive impact on the ambiance during the interview. Before the interviews were conducted, the first author was briefly introduced to the telehomecare services and the practical nurses working environment. The author's professional background and pre‐understanding of healthcare practices ensured a deeper mutual understanding, allowing for a more informed interpretation of the responses. The researcher was experienced in qualitative research methods and specifically digital interviews. To support credibility, the researcher avoided leading questions to decrease the impact of their own subjective opinions.

The analysis was performed by female researchers from various backgrounds, with experiences within caring sciences, nursing, sustainability and climate change. Four researchers analysed the data independently before performing joint in‐depth analyses. Confirmability of the research was achieved, as the researchers open‐mindedly discussed their own interpretations and welcomed each other's opinions. The researchers' potential bias was minimised by note‐taking and by having collaborative reflective discussions to maintain focus on the participants' experiences.

The research process has been thoroughly presented, to enhance transparency and dependability, making it possible for readers to determine if the study is applicable in other conditions. The research methods are described in detail, without falsification, to enhance transferability. Characteristics of participants, as well as inclusion and exclusion criteria, are mentioned in chapter 2.4.

## 3 Findings

**FIGURE 2:**
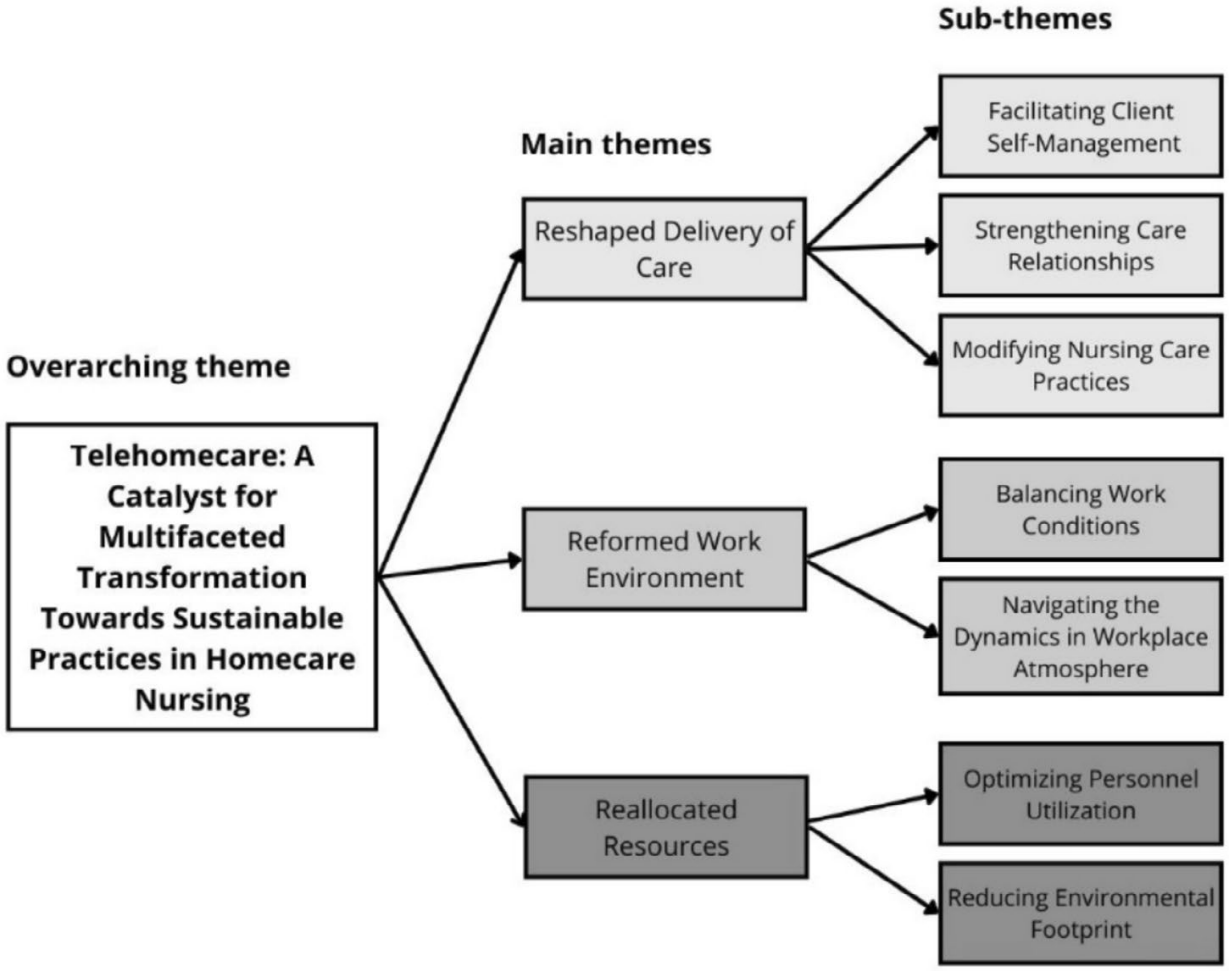
A presentation of themes describing how practical nurses experience the contribution of telehomecare to the transformation towards sustainable practices.

Based on the analysis, the overarching theme ‘Telehomecare: A catalyst for a multifaceted transformation toward sustainable practices in homecare nursing’ was developed. This theme described the shift in nursing in relation to the digitalisation of services and the need for more sustainable practices. This overarching theme consisted of the three main themes formed during the analysis, which had seven subthemes. The main themes were ‘Reshaped delivery of care’, ‘Reformed work environment’ and ‘Reallocated resources’ (Figure 2).

### 3.1 Reshaped Delivery of Care

#### 3.1.1 Facilitating Client Self‐Management

According to the participants, transferring care to a digital platform increased the accessibility of services, especially in rural areas. Additionally, the participants perceived that telehomecare enabled more timely care visits than the traditional physical homecare, under which visits were typically scheduled within a broader timeframe of several hours. The participants reported that clients tend to receive care from the same nurse more consistently in remote homecare settings than in physical visits. This continuity of care facilitated a more personalised approach to healthcare supervision, empowering clients to manage their health more independently. Furthermore, the participants described that care interactions through telehomecare could be concluded more discreetly and efficiently than physical homecare. One participant described the situation as follows:

P9: There are plenty of clients who do not want physical home visits. They prefer not to have anyone come over there and disturb them.

The participants described telehomecare as a valuable means of supporting clients to live at home for longer while promoting the independent initiative of clients. According to one participant, clients became more confident in their self‐management abilities. However, one participant feared that moving physical care to a digital platform might reduce physical contact with older people, who are already lonely:

P8: Maybe I am the only one who visits during the week. And for the rest of the time, they are alone, and we just call them.

According to the participants, telehomecare was most effective when clients were somewhat independent before the start of the service and did not have advanced care needs. Since telehomecare relied on active client participation, it also placed certain responsibilities on clients, such as ensuring that necessary devices, such as blood glucose meters, were functional and adequately powered. Additionally, the participants described some clients living at home as multimorbid and having care needs that required physical visits, making telehomecare a less suitable option for these individuals. Consequently, the participants highlighted the importance of introducing remote homecare services at earlier stages of old age before the progression of symptoms or illnesses required more intensive, physical interventions:

P2: […] Clients have been ill, not very ill, but they have perhaps been too ill for this service. Which has led to the telehomecare not being very long term, as they have not managed without physical visits.

Furthermore, technical challenges could adversely affect the quality of care and the overall client experience, according to the participants. A poor network connection could hinder both the delivery of care and the development of the nurse–client relationship. Background noise during video communication disrupted the care communication, and clients with hearing impairments may have had difficulty hearing personnel through the installed unit.

#### 3.1.2 Strengthening Care Relationships

The participants recognised the impacts of telehomecare on the relationship between nurse and client as well as on the care encounter. Telehomecare enabled remote visits from the same telehomecare nurse more frequently than was possible with physical homecare nurses, facilitating more effective communication and fostering a deeper mutual understanding. As nurses performing remote care did not have to do other tasks at the same time, such as household chores, telehomecare allowed them to be more present:

P1: The relationship is better in my opinion. We are really present. Clients say that it is just as if you were sitting at the coffee table. We do not do anything else in the background, we focus exclusively on the client and they say we actually listen […]. We do not have to do any chores at the same time.

All the participants expressed a deeper, interpersonal connection to clients with improved trust between both parties involved. The participants expressed that clients using telehomecare opened up to nurses in a different way than those using physical home care and recognised that clients seemed more comfortable sharing their problems:

P2: You become close in some way. Maybe it is because it is often the same nurse calling, they become comfortable and start trusting you, or maybe it is easier to cry in front of a camera, which is something you would not expect.

Furthermore, the participants expressed the importance of physical touch in care, recognising the need for both telehomecare and physical homecare visits. However, the participants presented other ways of showing compassion in a digital form by providing comfort and expressing emotional support:

P5: The physical touch is very important[…]. Of course, it is not quite the same but… We give good weekend wishes and long‐distance hugs, putting the clients in a really good mood.

#### 3.1.3 Modifying Nursing Care Practices

According to the participants, telehomecare had transformed their way of nursing. The participants described the risk of making clients passive by providing physical homecare, as practical nurses often faced time constraints during their visits. This limitation led them to prioritise efficiency, which could result in them performing tasks for clients rather than encouraging clients to engage in these activities themselves. In contrast to physical homecare, telehomecare required action from the client, making remote care more rehabilitative than physical homecare:

P8: It kind of helps clients to have more independent initiative. You do not wait for the nurse to come and inject insulin. Now when you make remote contact, they have to do it themselves.

However, all the participants expressed difficulties about conducting a comprehensive assessment of a client's well‐being remotely. The fixed positioning of cameras installed within clients' homes restricted the visual field, and nurses could only see what was directly in front of the camera. Consequently, practical nurses had to take the client at their word, as they could not observe the home environment and correlate verbal cues with physical actions. This limitation was particularly concerning in areas such as nutrition and hygiene, where physical observation was crucial:

P10: And this camera is pointed in one direction, so you only see where you…You do not really have an overview, like “have you eaten?” You cannot look for traces in the sink to see whether that is actually true.

Furthermore, the limited visual field in telehomecare proved to be particularly challenging for clients with conditions such as diabetes, as it increased the risk of incorrect insulin administration, potentially leading to harmful dosing errors:

P10: If they have, for example, insulin, they have to pull up the insulin pen and show us and know exactly how to do it. Sometimes you start thinking about safety, was it really, really right? If you are there physically you know exactly what they took and so on. It can be a little uncertain at times.

This limitation was especially critical in situations involving medication administration, such as verifying whether clients took their medication or administered injections correctly. While all the participants reported that medical situations were well managed overall, there remains a lack of confidence regarding clients' medication adherence:

P2: But you cannot be 100% sure that they have taken the medicine. That is, of course, a disadvantage. […] So it could be that they throw the pills into the trash… That is why I think it is really important to get that feeling of trust.

Telehomecare was described as supervision and guidance, underscoring the importance of nurses' competence, as they must possess the knowledge to ask the right questions and engage clients effectively. However, certain aspects of care, such as wound care and hygiene‐related tasks, remain challenging, if not impossible, to perform remotely due to the physical absence of the practical nurse in the client's home. In addition, practical nurses could not use all their senses to make an assessment, as one participant mentions:

P4: I think it is more challenging because here we cannot use all our senses when you go into a client's home. You notice by the smell or something that maybe the client has a urinary tract infection, or something else. But we cannot do it, we rely on what we see and hear. We observe.

### 3.2 Reformed Work Environment

#### 3.2.1 Balancing Work Conditions

The participants highlighted notable differences between working in physical and remote homecare. Performing telehomecare was described as a more comfortable and ergonomic way of working than physical homecare. Telehomecare was significantly less physically demanding than care performed face to face, making it possible for practical nurses with physical challenges to continue working in healthcare and potentially reducing their need for sick leave or early retirement. Furthermore, remote care enhanced personnel safety, as it eliminated the risk of exposure to inappropriate behaviour that could occur during physical home visits:

P7: Physical homecare has had a client […] that has been a bit touchy, so to speak. Especially with these female summer workers. So, they thought this remote visit would be a good alternative instead.

On the other hand, the participants also expressed concerns regarding the drawbacks of working digitally. Performing care digitally involved minimal physical activity, which resulted in physically passive working characterised by tedious, repetitive movements. Even though telehomecare makes for a physically passive working method, the participants expressed a demanding mental burden due to the prolonged emotional and psychological stress. According to the participants, the ease with which a client could end a telehomecare call fostered feelings of helplessness and powerlessness, highlighting the conflict between respecting the client's wishes and nurses' duty to protect clients' well‐being. One participant described the situation as follows:

P1: The client had really low blood sugar and he refused a physical visit from a nurse. He also forbade me to call him back later. Sometimes you just have to say “okay” and accept it, but it does bother you. It continues to bother you.

Furthermore, the participants emphasised the importance of adequate workspaces and technology to ensure a sustainable work environment. Most of the participants shared their workspace with multiple other nurses, and they expressed that the environment becomes acoustically challenging due to overlapping conversations. This not only hindered nurses from working undisturbed but also posed potential risks to client confidentiality. Additionally, the operation of equipment contributed to increased heat levels, which could adversely affect indoor air quality.

#### 3.2.2 Navigating the Dynamics in Workplace Atmosphere

The participants expressed that collaboration between nurses in the field and telehomecare nurses was needed when telehomecare was implemented as a service within the physical homecare setting. Some participants expressed the feeling of being left out of communication between physical homecare and registered nurses. Furthermore, the participants presented a rivalry between physical homecare and telehomecare nurses rooted in differing views on the value and nature of nursing work, particularly regarding whether clinical care and the emotional and communicative aspects of care were equally valuable. One participant described the situation as follows:

P1: Some nurses feel this is a bit boring and stupid. They feel that this is not nursing work and we feel that we are doing really good nursing work. We do the work that you do not get done, that is, we talk.

The participants described teamwork and a good work community as key to transforming ways of working. However, slow or inadequate development processes combined with nurses' inability to affect decisions on their working environment and their claims of not being taken seriously by the decision‐makers resulted in unsustainable working conditions:

P1: Working space solutions […], that is what we have asked for many years. We hope they can just do that, if nothing else. After all, if you work in peace, you feel good, then the client will feel good too. And then, we should take on more clients, but how do you take more clients when the space…

Inadequately coordinated work, especially if nurses made both physical and remote homecare visits, led to the staff not feeling at home anywhere. Not knowing what clothes to wear to work was described as ‘disastrous’, implying dissatisfaction with resource coordination and possible impacts on client interaction:

P2: It is very disastrous, because I do not know what to wear. How should I… You know, you go out, you get dressed, and then you come here and get changed and have to be relaxed and make video calls. You do not feel at home anywhere when it is like this.

### 3.3 Reallocated Resources

#### 3.3.1 Optimising Personnel Utilisation

The participants presented telehomecare as enabling more effective and sustainable homecare services, as personnel resources could be deployed more efficiently. Clients with demanding physical care needs could be prioritised in physical homecare, while telehomecare could relieve physical homecare nurses of interventions that could be done remotely. Furthermore, telehomecare could alleviate personnel shortages and make homecare services more efficient by reducing the visit peaks for physical care visits. When asked about the reasoning why remote homecare services were implemented in the well‐being services county, one participant responded:

P3: It was probably to streamline homecare in order to be able to reduce the peak visits that we have in the morning and evening […]. Maybe it is somewhat sustainable as well when you replace physical visits with digital visits. In that aspect we are a little more efficient.

The participants described telehomecare as more effective care work than physical homecare, making it more economically sustainable. As traditional home care relied on transportation by car, remote homecare visits could have an impact on travel for both personnel and clients. By performing care digitally, the personnel used less of their work time on travelling to the client as well as finding parking. When they no longer had to spend time travelling, practical nurses could make more care visits per day than were possible under physical homecare. The participants expressed that telehomecare reduced the clients' need for visits overall, as the clients' independence increased. The deeper relationship between nurse and client expressed by the participants, as well as frequent interactions with the same nurse, enabled nurses to observe and notice subtle changes in clients' behaviour effectively:

P2: You notice very quickly small things that have changed, which you might not notice on physical visits. We are a smaller team, so we see the clients almost daily. Whereas when you are out in the field, you do not. […] We suspected one had heart failure because we noticed symptoms during a digital visit, which the physical homecare did not notice, which later in the evening became acute. We often notice memory impairments, which come across very clearly.

Long‐term, sustainable planning of technical equipment was desired by practical nurses, as the installation of digital services took time and resources. The frequent replacement of devices within a few months due to changes in service agreements significantly impacted healthcare workflows. The process of reinstalling new equipment consumed valuable nursing time and disrupted nurses' work. Furthermore, inadequate technology caused additional work for the nurses and compromised the care experience, possibly impairing care visits completely, leading to clients not getting the care they need:

P4: The audibility is very bad, or the connection is bad in some parts of the country […], or we do not get a connection at all. […] If we do not get in touch via video call, we have to call them by phone. And that does not meet the criteria of why we are calling. Because we only rely on what the client says.

The participants said that plans to enhance sustainability and improve their working conditions were not carried out, and resources meant for development were withdrawn. Most of the practical nurses could not participate in the planning and procurement of technical equipment. When asked whether practical nurses could impact decisions regarding the technical aspects of the service, one participant replied:

P4: It feels like we cannot. It always has to go through someone else. There is someone who passes our requests forward, but there is never someone who takes action. […] It feels very, very much like it gets stuck in one place.

#### 3.3.2 Reducing Environmental Footprint

The participants recognised that telehomecare presented environmental benefits by eliminating the need for travel to clients' homes. This change in working practices was seen as climate‐friendly, as the reduced need for travel and use of vehicles directly contributed to decreased fuel consumption and associated environmental impacts. However, performing remote homecare visits required the use of technology, which increased electricity consumption both within healthcare organisations and in clients' homes, potentially raising electricity costs and the environmental effects related to electricity use:

P3: We leave no trash behind us. The only thing we use is electricity and the equipment.

Furthermore, telehomecare was described as enabling effective waste minimisation. Performing care remotely decreased the need for single‐use products, such as plastic gloves and plastic shoe covers, resulting in a reduction in waste generation and lower material costs. However, one participant raised concerns about the increase in electronic waste (e‐waste) associated with remote care:

P10: It is these machines later on, when they are to be destroyed of course. It is a general problem with that [e‐waste] for everyone.

The reduction in the use of single‐use materials was also associated with a decrease in the environmental guilt experienced by practical nurses. One participant specifically mentioned the feelings of shame they felt when they encountered plastic waste in natural environments, recognising it as a by‐product of physical homecare practices:

P1: We have these disposable shoe covers that physical homecare uses a lot. And it is noticeable, because you see shoe covers out in nature. It is annoying when you know exactly who they are from. A homecare worker!

## 4 Discussion

The aim of this study was to explore how practical nurses perceive telehomecare in relation to sustainability. The overarching theme of this study was “Telehomecare: A catalyst for a multifaceted transformation toward sustainable practices in homecare nursing,” with three main themes (Figure 2). The findings reveal that the participants have multifaceted perspectives, identifying that telehomecare has both negative and positive aspects in relation to sustainability. While the findings show that telehomecare promotes more sustainable nursing practices, it also introduced new complexities, highlighting the need for a comprehensive approach that considers all dimensions of sustainability, to achieve truly sustainable digital healthcare. In the following, the three main themes concerning the reshaped care process, reformed work environment and reallocated resources will be discussed. The main theme concerning the reshaped care process is examined through two distinct pathways: supporting client self‐management and streamlining service delivery.

### 4.1 Reshaped Care Process Supporting Client Self‐Management

The participants in this study indicated that telehomecare enhances healthcare accessibility, in line with the findings of Björndell and Prembeg (2021) and provides more rehabilitative benefits than traditional physical homecare, thereby contributing to the prevention of illness and supporting equal access to healthcare services. All these perceived advantages might, in turn, contribute to better patient outcomes, in line with the findings in a study by Orlando et al. (2019).

The participants consider telehomecare to be a more rehabilitative form of homecare, as it enables nurses to supervise and empower clients to manage their own needs, thereby fostering client independence and self‐management. This in turn contributes to an overall improvement in client well‐being and boosts the clients' confidence in their own abilities to self‐manage, possibly reducing their needs for visits overall, in line with the findings of Liang et al. (2021). Additionally, the clients' consistent contact with the same telehomecare nurse improved the caring relationship. This continuity fostered a greater sense of security and trust between the client and nurse, aligning with the findings of Hoffrén‐Mikkola et al. (2024). This finding emphasised the importance of a nurturing, holistic interaction to successfully deliver effective care at a distance supporting client self‐management. The participants perceived telehomecare as less intrusive to clients' privacy and autonomy, as care interactions can be concluded more discreetly and efficiently. However, the timely initiation of telehomecare services is critical, as the participants indicated that the service may not be suitable for clients with advanced care needs, as digital care also gives the client more responsibility.

The participants expressed concerns about the reduction of face‐to‐face interaction, particularly for an already socially isolated patient population. In the worst‐case scenario, patients may be deprived of a care visit entirely or partially due to technological limitations, further exacerbating these challenges and leading to patients not having the care they need. This possible outcome highlights a broader societal issue, as limited internet connectivity can be associated with the client's geographical location. The vulnerability of the healthcare system and services to technological limitations was also highlighted by Koltsida and Jonasson (2021).

### 4.2 Reshaped Care Process Streamlining Service Delivery

Aligning with previous research, this study highlights that telenursing may enhance the effectiveness and accessibility of care (Björndell and Premberg 2021), optimise personnel deployment (Muschol et al. 2022), minimise the use of single‐use products (Candel et al. 2021) and contribute to a more sustainable, longer‐lasting workforce. Furthermore, prior research highlights that telenursing reduces income loss for patients, as it minimises work absence (Muschol et al. 2022). This finding showed that telehomecare was not only beneficial in operational aspects of healthcare systems but also had socio‐economic value for clients by promoting health equity.

Telehomecare has demonstrated positive effects on various aspects of travel and contributes to the increased efficiency of nursing work. However, the participants reported uncertainty about assessments and medication adherence and concerns that patient safety might be compromised, in line with previous research (Koltsida and Jonasson 2021). These findings highlight a significant limitation that may undermine the quality and reliability of care delivery. Furthermore, some nursing tasks still require the physical presence of the nurse, highlighting additional limitations of remote care. It is essential to recognise that the effectiveness of telehomecare is dependent on the establishment of trust between nurses and patients. A lack of trust may result in double consultations, where patients utilise additional healthcare resources by seeking multiple consultations, thereby increasing both costs and environmental impacts. Additionally, there was a heightened risk of missed care in remote settings whereby care needs may go unnoticed and escalate into more severe conditions if not addressed promptly. Missed care negatively impacts patient outcomes, quality, safety and satisfaction (Hopkins Walsh and Dillard‐Wright 2020).

Considering nursing practice, the findings of this study indicate that telehomecare facilitates an enhanced form of caring with a deeper client–nurse relationship. A contributing factor to this improvement is likely the continuity of care: clients receive frequent visits from the same nurse, which enables the nurses to detect subtle changes in their health and well‐being. The increased mental presence of the nurse supports a strengthened interpersonal relationship, which, in turn, enhances the sense of trust between the client and the nurse, making clients more likely to share their concerns, thus supporting the findings of Hoffrén‐Mikkola et al. (2024). However, this improvement in relationships contradicts the findings of Knop et al. (2024), who saw technology as a barrier to good interaction and patient‐centred care. This contradiction shows the complexity of care and suggests that the impact of technology on caring relationships is not consistent. Given that telehomecare depends heavily on the active participation of clients, the nurses' ability to engage clients effectively and collect comprehensive client information is of critical importance also indicated by previous research (Björndell and Premberg 2021). Furthermore, telehomecare allows for more timely care interventions and enables nurses to remain fully focused on client care, as they are not distracted by the need to undertake chores in the clients' homes. However, the participants highlighted the lack of physical touch that is vital in nursing care and recognised the need for traditional homecare in addition to telehomecare visits, in line with previous studies (Knop et al. 2024). The lack of physical touch needed in care is compensated by digital ways of showing compassion, for example, by giving hugs at a distance. However, the dual provision of digital and in‐person care may place additional demands on healthcare resources, potentially undermining efforts to achieve economic sustainability in service delivery.

The findings underscore several technical challenges that impede the seamless delivery of telehomecare services, supporting the findings of Smith et al. (2020) and Koltsida and Jonasson (2021), stressing the importance of adequate technical infrastructure. Inadequate technology and unreliable internet connectivity were identified as significant barriers affecting both the delivery and quality of care, raising ethical concerns about patients' rights to healthcare. Concerns regarding confidentiality and data security were raised, as the use of technology in remote care introduces certain risks to privacy. Moreover, the unsustainable planning of technology implementation further disrupts workflow. Therefore, long‐term planning before the installation of devices is crucial to ensure the sustained use of equipment over several years rather than in short‐term cycles. The findings might indicate a need for additional innovative technological solutions to ensure secure nursing situations and adequate assessments.

### 4.3 Reallocated Resources Reducing Climate Impact

The study was conducted within a well‐being services county that encompasses both rural and urban regions and is characterised by significant distances to healthcare centres. In this context, telehomecare emerges as a viable alternative with considerable potential to alleviate environmental impacts. In line with previous research, the participants highlighted that nursing care performed remotely could significantly reduce the organisation's environmental footprint by decreasing vehicle use, fuel consumption and associated costs (Donald and Irukulla 2022; Knop et al., 2021), and the use of single‐use materials, thus contributing to lower waste production and material costs (Candel et al. 2021). No participant in this study mentioned public transportation as an optional form of travel, possibly because of the inadequate infrastructure in the area where the study was performed. Furthermore, the participants acknowledged that telehomecare leads to increased electricity consumption (Holmner et al. 2014) for both clients and healthcare providers, aligning with findings from prior research, contributing to the rising issue posed by the rapid surge in global energy consumption.

The findings highlight that the technology required for telehomecare eventually results in e‐waste when the technology is disposed of, contributing to the problem of e‐waste as well as the rising need for minerals for technological development. Additionally, previous research has emphasised the potential of digital healthcare to reduce the health risks associated with road traffic (Purohit et al. 2021). Furthermore, it is worth noting that practical nurses undertaking physical homecare are often poorly compensated and must use their own vehicles in their work, typically powered by gasoline, delaying the transition to more sustainable practices.

### 4.4 Reformed Work Environment Supporting a More Sustainable Workforce

An important aspect that is not fully addressed in the principles of sustainable healthcare by Mortimer (2010) is the well‐being of healthcare personnel. With unsustainable working conditions within healthcare systems worldwide, it is important to prioritise and enhance the well‐being of healthcare personnel to support workforce retention (Giordano et al. 2023). The findings in this study suggest that telehomecare may support workforce retention by reducing sick leave and preventing early retirement due to injury.

According to Björndell and Premberg (2021), remote nursing technologies support personnel well‐being and work situations, suggesting telehomecare is a sustainable work form. The findings in this study indicate that telehomecare makes for a more sustainable workforce and inclusive work environment by enabling nurses with physical challenges to extend their work life, aligning with the findings of Hoffrén‐Mikkola et al. (2024). Telehomecare is considered a more comfortable and safer working method than traditional physical homecare, as it makes fewer physical demands. However, these benefits are somewhat limited by the repetitive nature of telehomecare tasks, risk of passive working and increased mental strain. Inadequate workspaces and technology increase the distractions within the work environment, possibly adding stress. Telehomecare also protects nurses from physically inappropriate patient behaviour that negatively impacts their well‐being. Furthermore, the reduction in physical home visits may alleviate environmental concerns, such as the potential risk of plastic waste in natural settings, thereby lessening climate‐related stress and guilt among nurses, a phenomenon becoming more frequent among young people (Brophy et al. 2023). Additionally, reducing the use of plastics minimises possible uncharted health risks related to microplastics and chemicals (Rodrigues et al. 2019). These findings show that telehomecare can transform nursing work by promoting sustainability and well‐being, while also revealing areas of improvement in digital work environments.

Collaboration and teamwork are highlighted as critical components of sustainable healthcare practices, aligning with previous research (Cruz et al. 2024), to drive a collective transformation towards more sustainable practices. The findings reveal a lack of effective collaboration and mutual understanding between telehomecare and physical homecare nurses. This aligns with the study by Knop et al. (2021), where varying levels of digital competence result in an imbalance of power between nurses. On the other hand, digital tools facilitate accessible collaboration (Koltsida and Jonasson 2021). Furthermore, the practical nurses reported limited involvement in organisational decision‐making processes and insufficient resources for development. These factors foster resistance among personnel and a division in the organisation's workforce. Inadequate resource coordination leads to stress, leaving nurses feeling disconnected and lacking a sense of belonging within their work environments. This aligns with previous studies, where nurses' perceived ability to influence their work situation was strongly related to their satisfaction with telehomecare (Hoffrén‐Mikkola et al. 2024), highlighting the importance of nurse involvement, contributing to the sustainability of the healthcare workforce.

### 4.5 Strengths and Limitations of the Work

This study was performed in Finland, with participants from one well‐being services county. This might be seen as a limitation to the study. As homecare services, healthcare systems and ‐delivery vary in different countries, this limits the applicability of the findings. Furthermore, it is important to emphasise that the study examines the perceptions of caregivers, rather than patients, which restricts the applicability of the findings. All the participants were female, no male participants being applicable for this study, which presents a limitation to the study findings. The sample size of 10 was deemed sufficient, as information power was achieved.

The concept of sustainability was introduced to the participants prior to the interviews, possibly influencing the study results. The first author had extensive clinical experience as a practical nurse and registered nurse in the field of physical homecare. This added a strength to the study, as the researcher had a broad understanding of the nursing tasks and working conditions. However, it is important to note the subjective nature of thematic analysis, as the findings might be unintentionally influenced by the researchers' bias.

The participants could choose to be interviewed face to face or digital. All the participants chose to be interviewed digitally, which is both a strength and a limitation. Digital interviews result in more reflective responses and mitigate resources used for travel, but also hinder contextual aspects such as non‐verbal communication and the researcher's ability to create a positive ambiance (Deakin and Wakefield 2014). The researcher was experienced in digital interviews, which added to the reliability of the study. Furthermore, it is recommended to perform research in the participants' natural environment, which makes digital interviews preferred as all the participants worked in the digital milieu.

## 5 Conclusion

In this study, the participants presented sustainability‐related perceptions of telehomecare. This novel research area indicates that telehomecare is transforming nursing by reshaping the delivery of care, reforming the work environment and reallocating resources. This finding demonstrates telehomecare may be an effective option for more sustainable healthcare practices, although having notable limitations. The findings suggest that telehomecare reduces the carbon footprint of healthcare organisations from a nursing care perspective while simultaneously introducing challenges related to workplace environment and dynamics and nursing practices, among other factors.

There is a need for a holistic approach to sustainable nursing care practices in digital forms of healthcare, pinpointing social, economic and environmental aspects while taking into account the possible consequences and rebound effects of the sustainable digital healthcare transformation. Financial investment is essential to work towards more sustainable healthcare, aligning with this study's contribution to the ongoing discourse on the healthcare sector's environmental responsibility and the ethical aspect of ensuring equitable and adequate healthcare both now and in the future. The findings in this study indicate the need for comprehensive education and training in sustainable digital work practices for healthcare professionals, leaders and policymakers to strengthen knowledge and awareness as well as more sustainable coordination and collaboration within healthcare organisations. Transformative leadership is needed to drive organisational change towards more sustainable healthcare practices in everyday client interactions and to implement effective strategies and financial investments in digital sustainability that respond to the rising challenges in the social, economic and environmental aspects of healthcare.

### 5.1 Recommendations for Further Research

The participants in this study were all female, adults, practical nurses with experience in telehomecare visits and/or care services for older adults. Therefore, further research is recommended on a more heterogeneous sample. Furthermore, future research could focus on other types of technology, such as robotics or AI‐driven tools, to widen the perspective on telenursing and sustainability. As sustainable practices are essential throughout the healthcare context, research on other stakeholders, such as clients, physicians and unofficial caregivers, could enhance the knowledge on future sustainable healthcare. In addition, digital care and digital caring communication are research areas that could be strengthened and be of value to society in the process of digitisation of healthcare services.

### 5.2 Implications for Policy and Practice

This novel research area indicates that telehomecare is transforming nursing by reshaping the delivery of care, reforming the work environment and reallocating resources. Its findings indicate the social, economic and environmental aspects that must be accounted for when telehomecare services are implemented to work towards more sustainable homecare from a nursing care perspective. Comprehensive policies and strategies on digital sustainability are needed to navigate this paradigm shift, as well as training and education on sustainable nursing activities in the digital context. Strengthened transformative leadership is essential to drive effective changes in practices aimed at reducing environmental impact. Such leadership should foster a collaborative work environment and enhance communication to improve overall workplace conditions. Additionally, financial investments, as well as advancements in technology and improved infrastructure, are crucial to successfully achieving these goals and working towards more sustainable healthcare.

## Author Contributions

R.M., A.M., A.A., E.L. and F.L. made substantial contributions to conception and design, or acquisition of data, or analysis and interpretation of data; R.M., A.M., A.A., E.L. and F.L. Involved in drafting the manuscript or revising it critically for important intellectual content; R.M., A.M., A.A., E.L. and F.L. Given final approval of the version to be published. Each author should have participated sufficiently in the work to take public responsibility for appropriate portions of the content; R.M., A.M., A.A., E.L. and F.L. Agreed to be accountable for all aspects of the work in ensuring that questions related to the accuracy or integrity of any part of the work are appropriately investigated and resolved.

## Conflicts of Interest

The authors declare no conflicts of interest.

## Data Availability

The data that support the findings of this study are available from the corresponding author upon reasonable request.
